# The focal adhesion protein Testin modulates KCNE2 potassium channel β subunit activity

**DOI:** 10.1080/19336950.2021.1874119

**Published:** 2021-01-19

**Authors:** Maria Papanikolaou, Shawn M. Crump, Geoffrey W. Abbott

**Affiliations:** Bioelectricity Laboratory, Department of Physiology and Biophysics, School of Medicine, University of California, Irvine, CA, USA

**Keywords:** KCNQ1, KCNA5, voltage-gated potassium channel, coronary artery disease

## Abstract

Coronary Artery Disease (CAD) typically kills more people globally each year than any other single cause of death. A better understanding of genetic predisposition to CAD and the underlying mechanisms will help to identify those most at risk and contribute to improved therapeutic approaches. KCNE2 is a functionally versatile, ubiquitously expressed potassium channel β subunit associated with CAD and cardiac arrhythmia susceptibility in humans and mice. Here, to identify novel KCNE2 interaction partners, we employed yeast two-hybrid screening of adult and fetal human heart libraries using the KCNE2 intracellular C-terminal domain as bait. Testin (encoded by *TES*), an endothelial cell-expressed, CAD-associated, focal adhesion protein, was identified as a high-confidence interaction partner for KCNE2. We confirmed physical association between KCNE2 and Testin *in vitro* by co-immunoprecipitation. Whole-cell patch clamp electrophysiology revealed that KCNE2 negative-shifts the voltage dependence and increases the rate of activation of the endothelial cell and cardiomyocyte-expressed Kv channel α subunit, Kv1.5 in CHO cells, whereas Testin did not alter Kv1.5 function. However, Testin nullified KCNE2 effects on Kv1.5 voltage dependence and gating kinetics. In contrast, Testin did not prevent KCNE2 regulation of KCNQ1 gating. The data identify a novel role for Testin as a tertiary ion channel regulatory protein. Future studies will address the potential role for KCNE2-Testin interactions in arterial and myocyte physiology and CAD.

## Introduction

Coronary Artery Disease (CAD) typically leads to more deaths in the US. and globally each year than any other single cause of death [1]. Around half of CAD cases involve genetic predisposition, while reduction of other risk factors could reduce CAD mortality and morbidity by >30% [2]. A major challenge is to develop more comprehensive prevention and treatment strategies for both genetic and environmental risk factors [2]. This will require a fuller mechanistic understanding of CAD. Another form of fatal cardiac event, Sudden Cardiac Death (SCD), kills 1000 people per day in the U.S.. SCD probably requires electric and ischemic substrates, and a trigger [3]. Despite recent advances in our understanding of SCD, there is still much to learn. Most of the 25 genes linked to SCD also serve roles outside the heart, therefore it makes sense to consider how disruption of these extracardiac functions influences SCD and arrhythmogenesis, and in some cases also CAD. Many SCD-linked genes encode ion channel pore-forming (α) subunits, but the remainder encode proteins that regulate them [3,4]. It is advantageous to understand the biology and pathobiology of the entire macromolecular ion channel complex.

KCNE subunits are single-transmembrane domain proteins that form complexes with voltage-gated potassium (Kv) channel α subunits to alter all aspects of their function and biology. *KCNE* genes are associated with human cardiac arrhythmias including Long QT Syndrome (LQTS), Brugada Syndrome (BrS) and atrial fibrillation (AF) [5,6], and KCNEs serve broad roles extending beyond direct regulation of Kv channel electrical attributes [7–14]. Studies of *Kcne* knockout (^–/–^) mice have predicted disorders that were subsequently linked to human gene disruption [15–19]. We and others recently discovered an unexpected link between KCNE2 and CAD, in humans [20,21] and in mice [22]. Aside from *Kcne2*, only two other single-gene knockouts (*ApoB* and *Ldlr*) induce atherosclerosis in mice [23]. Beyond this, *Kcne2* gene deletion causes a multisystem syndrome predisposing to SCD, which includes multiple risk factors for CAD, including diabetes, elevated serum LDL and angiotensin II (Ang II). The spectrum of *Kcne2* disruption-linked disorders in mice therefore provides multiple electric and ischemic substrates, and even a trigger (fasting-induced hypoglycemia) for SCD [24]. Aspects of this KCNE2-linked multisystem pathology have emerged in human population studies [20,25].

Here, to pursue a fuller understanding of the mechanisms of action of KCNE2 and its roles in cardiovascular disease, we conducted yeast two-hybrid screens to identify novel interacting partners expressed in human heart tissue. The screen identified a high-confidence hit, Testin, which is a CAD-associated focal adhesion protein. We confirm its physical interaction with KCNE2 and demonstrate its ability to alter the functional attributes of Kv channel complexes.

## Methods

### Yeast two-hybrid

Yeast two-hybrid screens were conducted by Hybrigenics Services, S.A.S. (Paris, France). The coding sequence for Homo sapiens – KCNE2 (aa 66–123) (GenBank accession number gi: 27436977) was amplified by PCR and cloned into pB27 as a C-terminal fusion to LexA (N-LexA-KCNE2-C) and into pB66 as a C-terminal fusion to Gal4 DNA-binding domain (N-Gal4-KCNE2-C). Construct integrity was verified by sequencing. The constructs were used as bait to screen a random-primed human ventricle and embryo heart cDNA library constructed into pP6. pB27, pB66 and pP6 derive from the original pBTM116 [26], pAS2ΔΔ[27] and pGADGH [28] plasmids, respectively.

For the LexA bait construct, 145 million clones (18-fold the complexity of the library) were screened using a mating approach with YHGX13 (Y187 ade2-101::loxP-kanMX-loxP, matα) and L40ΔGal4 (mata) yeast strains as previously described [27]. A total of 33 His+ colonies were selected on a medium lacking tryptophan, leucine and histidine, and supplemented with 2 mM 3-aminotriazole to handle bait autoactivation. For the Gal4 construct, 66 million clones (eightfold the complexity of the library) were screened using the same mating approach with HGX13 (Y187 ade2-101::loxP-kanMX-loxP, matα) and CG1945 (mata) yeast strains. A total of 37 His+ colonies were selected on a medium lacking tryptophan, leucine and histidine, and supplemented with 0.5 mM 3-aminotriazole to handle bait autoactivation. The prey fragments of the positive clones were amplified by PCR and sequenced at their 5ʹ and 3ʹ junctions. The resulting sequences were used to identify the corresponding interacting proteins in the GenBank database (NCBI) using a fully automated procedure. A confidence score (Predicted Biological Score) was attributed to each interaction as previously described [29]. The Predicted Biological Score relies on two different levels of analysis. First, a local score includes consideration of the redundancy and independency of prey fragments, in addition to the distribution of reading frames and stop codons in overlapping fragments. Second, a global score takes into account the interactions found in all the screens performed at *Hybrigenics* using the same library. The global score indicates the probability of an interaction being nonspecific. For practical use, the Predicted Biological Scores are divided into six categories, including A (highest confidence) to D (lowest confidence). A fifth category (E) specifically flags interactions involving highly connected prey domains previously found several times in screens performed on libraries derived from the same organism. Finally, several of these highly connected domains have been confirmed as false positives of the technique and are designated as F. Importantly, the Predicted Biological Scores positively correlate with the biological significance of interactions [30].

### Cell culture and transfection

We seeded CHO cells (ATCC) onto poly-L-lysine treated glass coverslips and transfected using TransIT-LT1 (Mirus Bio LLC, Madison, WI, USA) the following day with CMV-based expression constructs containing cDNA for human TES (DDK-tagged), KCNE2 (HA or mCherry-tagged), KCNA5 (CFP-tagged), and/or KCNQ1 (GFP-tagged for electrophysiology). Cells were cultured in DMEM with 10% FBS and 1% penicillin/streptomycin in a 95% O_2_/5% CO_2_ humidified environment at 37°C for 48–72 hours post-transfection prior to biochemical analysis, imaging or patch-clamping. We purchased cell culture consumables and reagents from VWR or Fisher Scientific unless otherwise stated.

### Protein biochemistry

For co-immunoprecipitations, we first pre-cleared all samples by incubating the total CHO cell lysate with protein A/G PLUS-coated agarose beads (Santa Cruz) for 1 h. Beads were then pelleted and discarded. Total protein was quantified by BCA. We then added immunoprecipitating antibodies at a dilution of 1:100 for overnight pulldown at 4°C. The following day, we immunoprecipitated antibody-antigen complexes with fresh protein A/G PLUS agarose beads (Santz Cruz biotechnology, Dallas, TX), prior to western blotting. For western blotting, we conducted SDS-PAGE, and then transferred proteins onto PVDF membranes for immunoblotting with the following primary antibodies: mouse anti-DDK (Santa Cruz Biotechnology), rabbit anti-HA (Santa Cruz Biotechnology). For secondary detection, we used horseradish peroxidase (HRP)-conjugated antibodies (BioRad, Hercules, CA) in conjugation with Luminata Forte HRP substrate (Millipore Sigma, Burlington, MA). We imaged the western blots using Gbox hardware and software (Syngene, Cambridge, UK).

### Whole-cell patch clamp

We recorded currents expressed in CHO cell using whole-cell patch-clamp at room temperature (22–25°C) with 3–6 MΩ borosilicate glass electrodes backfilled with solution containing (in mM): 90 K Acetate, 20 KCl, 40 HEPES, 3 MgCl_2_, 1 CaCl_2_, 3 EGTA-KOH, 2 MgATP; pH7.2. We perfused cells continuously at 1–2 ml/min with extracellular solution containing (in mM): 135 NaCl, 5 KCl, 5 HEPES, 1.2 MgCl_2_, 2.5 CaCl_2_, 10 glucose; pH 7.4. We purchased chemicals from Fisher Scientific or Sigma-Millipore. We held cells at −80 mV in voltage clamp before applying the voltage step protocols and recording currents in response to pulses between −80 mV and +40 or +60 mV at 10 or 20 mV intervals, followed by a single pulse to −30 mV, using a CV −7A Headstage (Axon Instruments, Foster City, CA, USA). Currents were amplified using a Multi-clamp 700B (Axon Instruments), low-pass filtered at 2–10 kHz using an eight-pole Bessel filter and digitization was achieved (sampling at 10–40 kHz) through a DigiData 1322A interface (Molecular Devices; Sunnyvale, CA). We used pClamp8 (Molecular Devices) Clampex software for data acquisition and Clampfit software for analysis, together with Graphpad Prism 7.0 (Graphpad; La Jolla, CA, USA). We plotted normalized tail currents versus pre-pulse voltage and fitted with a single Boltzmann function to examine voltage dependence.

### Statistical analysis

All values are expressed as mean ± SEM. Students’ t-test was used for statistical comparisons. All P-values were two-sided. Statistical significance was defined as P < 0.05.

## Results

### KCNE2 physically interacts with Testin

**Figure 1.**
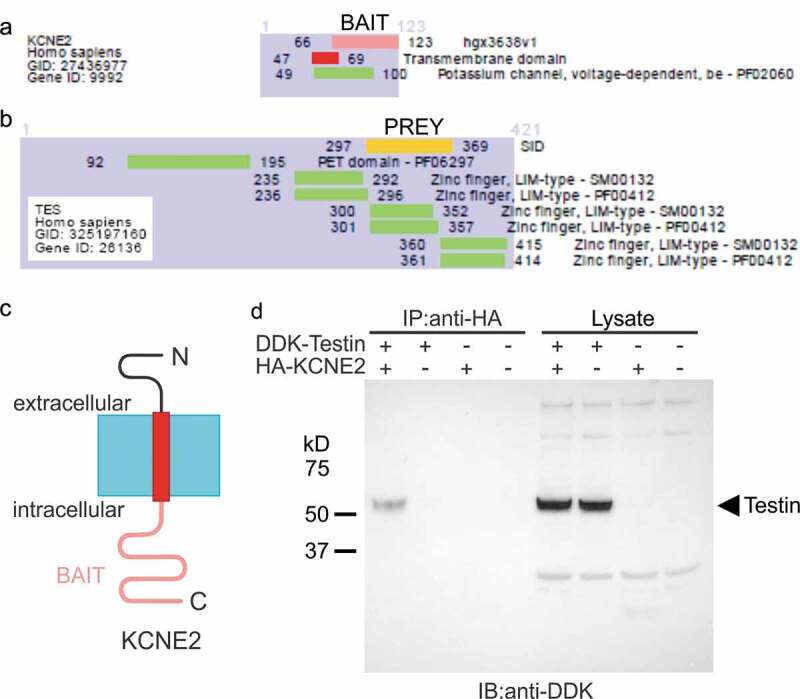
KCNE2 forms complexes with Testin. (a,b) Summary of yeast two-hybrid results indicating the region of Testin (residues 297–369, in yellow) (PREY) common to all clones interacting with the KCNE2 C-terminal domain (residues 66–123, in pink) (BAIT). SID = Selected Interacting Domain

Several interactions with the KCNE2 C-terminal domain were discovered by yeast two-hybrid screening. Among them, KCNE2-Testin interaction was detected with six independent clones and using both screening systems, the LexA and the Gal4. The KCNE2-Testin interaction was ranked with a high-confidence score (Predicted Biological Score = B). The common sequence of the six independent clones incorporated Testin residues 297–369 and contains the zinc finger, LIM-type functional domain (Figure 1(a–c)). Co-immunoprecipitation studies in CHO cells expressing epitope-tagged versions of KCNE2 and Testin confirmed that the two proteins closely associate in the absence of any Kv α subunit partners (Figure 1(d)). Interestingly, Testin, encoded by *TES*, is a focal adhesion protein [31] that has been shown to be important for endothelial cell integrity, and which was found to be sixfold downregulated in humans with CAD compared to controls [32]. Therefore, KCNE2 and Testin are each linked to similar pathophysiology, although support for a common mechanism underlying this will require future studies in native tissue or animals, beyond the scope of the present manuscript.

(c). Transmembrane topology of KCNE2 showing the intracellular portion used for bait in yeast two-hybrid (pink). (d) Representative western immunoblot (IB) (from *n* = 2) confirming physical association by co-immunoprecipitation (IP) of DDK-tagged Testin with HA-tagged KCNE2 alongside IP controls; lysate controls are shown on right.

### Testin prevents regulation of Kv1.5 voltage dependence by KCNE2

**Figure 2.**
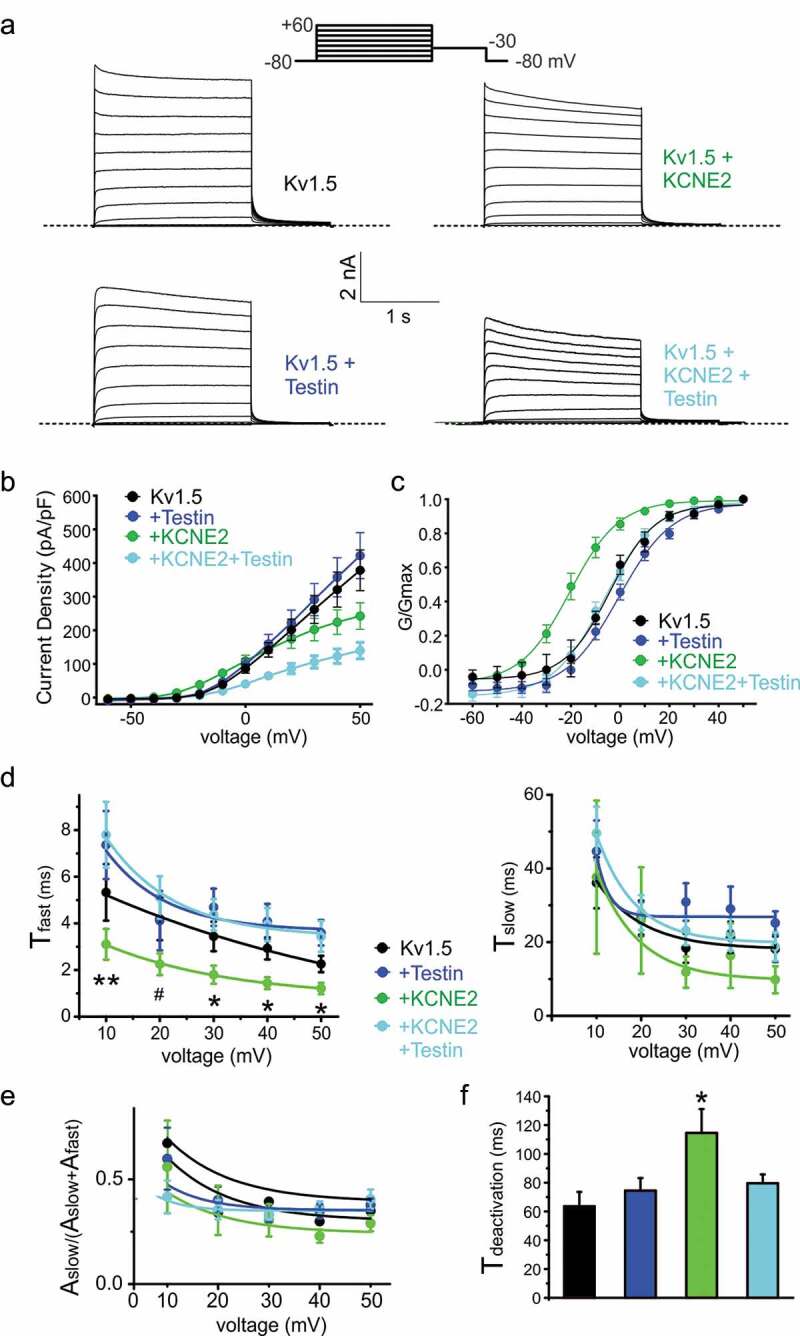
Testin modulates KCNE2 effects on Kv1.5

Kv1.5 is a voltage potassium channel (Kv) α subunit expressed in atrial myocytes and endothelial cells [33]. We previously showed that *Kcne2* deletion in mice impairs ventricular myocyte Kv1.5 (KCNA5) currents [34]. Here, we show that human KCNE2 negative-shifts the voltage dependence of Kv1.5 activation by ~-20 mV, greatly increasing the ability of Kv1.5 to open at subthreshold membrane potentials, but reduces peak current density at depolarized membrane potentials by 40% (Figure 2(a–c)). Co-expression of Testin had no effect on Kv1.5 voltage dependence or current density in the absence of KCNE2. However, co-expression of all three proteins resulted in elimination by Testin of the negative shift in Kv1.5 voltage dependence induced by KCNE2, and a 60% reduction in current density compared to Kv1.5 alone (Figure 2(a–c)).

All error bars indicate SEM. (a) Exemplar traces showing whole-cell patch-clamp recordings from CHO cells transfected with the subunit combinations shown. Dotted line indicates zero current level. Upper inset shows the voltage protocol.(b) Mean current density for currents expressed by subunit combinations indicated. Kv1.5, *n* = 13; Kv1.5 + Testin, *n* = 14; Kv1.5 + KCNE2, *n* = 15; Kv1.5 + Testin + KCNE2, *n* = 19.(c) Mean G/Gmax calculated from tail current for cells as in B.(d) Mean activation rate quantified as fast (T_fast_) and slow (T_slow_) components from a double exponential fit, for cells as in B. * P < 0.05; ** P < 0.01 for Kv1.5 + KCNE2 versus other groups; # P < 0.05 for Kv1.5 + KCNE2 versus Kv1.5 + KCNE2 + Testin. (e) Relative amplitude of the slow component of activation for cells as in B. Mean deactivation rate quantified as T of a single exponential fit, for cells as in B.* P < 0.05 versus other groups.

### Testin prevents regulation of Kv1.5 gating kinetics by KCNE2

We assessed Kv1.5 activation rate by fitting with a double exponential function. KCNE2 increased the rate of Kv1.5 activation, specifically by accelerating the fast component of activation (Figure 2(d,e)). In contrast, Testin had no effect on Kv1.5 activation rate. Furthermore, Testin eliminated the effects of KCNE2 on Kv1.5 activation rate when the three were co-expressed (Figure 2(d,e)). Correspondingly, KCNE2 slowed Kv1.5 deactivation by >40%, while Testin had no effect alone on Kv1.5 deactivation rate. Again, Testin eliminated the effects of KCNE2 on Kv1.5 deactivation rate (Figure 2(f)).

### Testin does not alter KCNE2 regulation of KCNQ1

**Figure 3.**
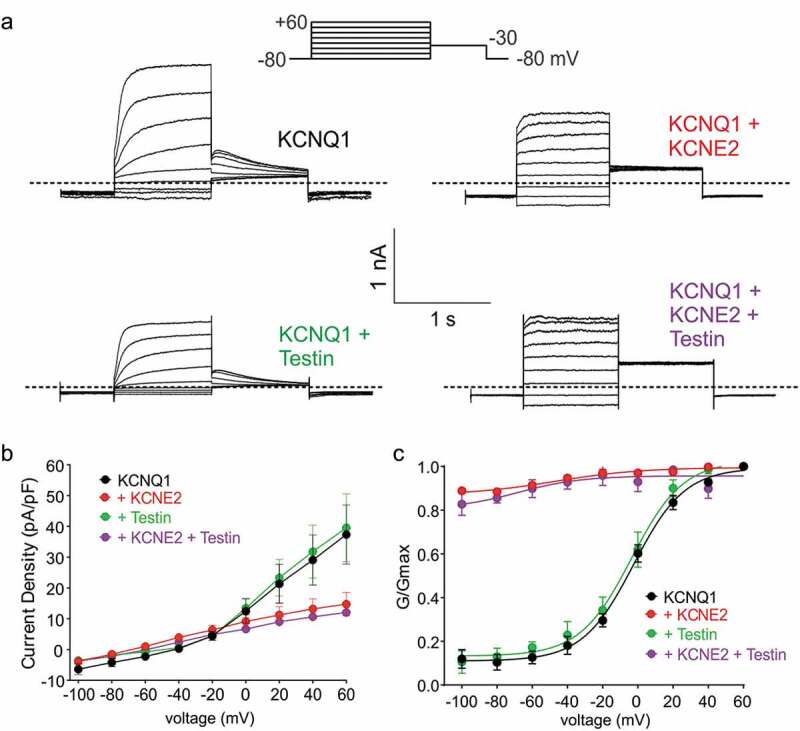
Testin does not alter KCNE2 modulation of KCNQ1

KCNE2 regulates many other Kv channel α subunits, including KCNQ1 (Kv7.1). KCNQ1-KCNE2 complexes are constitutively active K^+^ channels that generate relatively small (compared to homomeric KCNQ1) K^+^ currents with a linear voltage dependence, in gastric parietal cells, thyroid cells, choroid plexus epithelium and probably pancreatic β cells [16,35–37]. Here, we recapitulated published effects of KCNE2 on KCNQ1 using CHO cell expression and whole-cell patch-clamp (Figure 3(a)). Testin had no effects on KCNQ1 voltage dependence or current density; neither did it alter KCNE2 regulation of KCNQ1 (Figure 3(a–c)). Thus, Testin modulation of KCNE2 function is specific to the α subunit that KCNE2 is regulating.

All error bars indicate SEM. (a) Exemplar traces showing whole-cell patch-clamp recordings from CHO cells transfected with the subunit combinations shown. Dotted line indicates zero current level. Upper inset shows the voltage protocol. (b) Mean current density for currents expressed by subunit combinations indicated. KCNQ1, *n* = 14; KCNQ1 + Testin, *n* = 12; KCNQ1 + KCNE2, *n* = 12; KCNQ1 + Testin + KCNE2, *n* = 13.(c) Mean G/Gmax calculated from tail current for cells as in B.

## Discussion

After the original report linking a SNP near the human *KCNE2* locus to early-onset myocardial infarction (MI) [25], a different SNP within the human *KCNE2* gene itself was reported to be associated with predisposition to CAD [20], and related findings have followed [38,39]. These discoveries suggested a link between human KCNE2 and CAD, but functional and mechanistic data were lacking. We recently established causality by finding that *Kcne2* deletion in mice predisposes to atherosclerosis [22]. The *Kcne2*^–/–^ mouse data supported the association between CAD and KCNE2 observed in human studies, and are notable because mouse atherosclerosis models are rare and not previously associated with ion channel subunits [40]. *Kcne2*^–/ –^ mice exhibit many risk factors for CAD [22,24,41], but a precise molecular mechanism for Kcne2-linked CAD is lacking. It is therefore of interest that Testin is now identified, in an unbiased yeast two-hybrid screen, as a high-confidence interaction partner for KCNE2.

Testin is a focal adhesion protein that functions in cell motility and adhesion [31]. Strikingly, Testin was previously found to be sixfold down-regulated in the coronary arteries of people with CAD [32]. Testin is required for endothelial integrity and its disruption promotes trans-endothelial migration of monocytes, facilitating CAD [32]. The KCNE2-interacting fragments in our screens overlapped with the Testin LIM2 domain, consistent with the established role of LIM domains in protein–protein interactions [42]. Also of interest, Testin is a tumor suppressor gene, and when challenged with a carcinogen, Testin null mice are predisposed to developing gastric cancer [43]. This increases the pathophysiological overlap of Testin with KCNE2, because *Kcne2* null mice spontaneously develop gastric cancer [17]. KCNQ1-KCNE2 complexes are essential for gastric acid secretion through the parietal cell H^+^/K^+^-ATPase [15,16,44]. Germline *Kcne2* disruption causes achlorhydria, gastric hyperplasia, gastritis cystica profunda, adenomatous polyps and gastric metaplasia [17]. While bacterial overgrowth arising from achlorhydria can lead to inflammation and potentially predispose to metaplasia, *Kcne2* disruption also causes potentially carcinogenic cell cycle changes at the cellular level and is associated with increased cancer cell migration in vitro, independent of changes to stomach pH and tissue inflammation [45].

With respect to the implications of the effects of Testin on KCNE2 regulation of Kv1.5, we previously found that KCNE2 is required for normal Kv1.5 activity in ventricular myocytes; *Kcne2* knockout in mice resulted in reduction in Kv1.5 current (and susceptibility to drug-induced LQTS) because without *Kcne2*, Kv1.5 trafficking to the intercalated discs was disrupted [34]. It will be of interest in future studies to determine whether Testin association with KCNE2 reroutes trafficking of channels incorporating these subunits, or if Testin knockout mice exhibit cardiac electrophysiological abnormalities. In endothelial cells of the vasculature, downregulation of Kv1.5 resulted in membrane depolarization and decreased endothelium-dependent relaxation to acetylcholine, in a model of pulmonary artery hypertension [46]. Furthermore, loss of Kv1.5 vasomotor function was previously proposed to contribute to microvascular dysfunction in CAD and other vascular diseases, based on the observation that impairment of H_2_O_2_-induced dilation in CAD was associated with loss of Kv1.5 expression [47]. Future work can be directed toward understanding the potential role of KCNE2 and Testin in these effects, and whether they are required for the normal function of Kv1.5 in vascular endothelial cells.

### Conclusions and Limitations

Increasing evidence demonstrates that KCNE effects are not limited to their effects on channel gating or cellular localization. For example, KCNE1 and KCNE2, but not KCNE3, transduce the functional effects of KCNQ1 phosphorylation (which also requires Yotiao), facilitating regulation of cardiac KCNQ1 activity by the sympathetic nervous system [48,49]. In addition, we found that KCNE1 is required for PKC-sensitive endocytosis of KCNQ1 [11], explaining previously discovered effects of PKC on I_Ks_ [50,51]; and that KCNE2 reverses the effects of KCNQ1 on SMIT1 activity [52]. These and other findings, together with our current observations for Testin, suggest KCNE proteins augment the bidirectional signaling capacity of ion channel complexes.

There are several limitations to the present study, suggesting additional lines of experimentation to be pursued in the future. First, we did not investigate native KCNE2-Testin protein–protein interaction, due to a current lack of Testin antibodies of sufficient quality to conduct these studies rigorously. Thus, we do not yet know the native physiological relevance of the KCNE2-Testin interaction. Second, while we demonstrate that heterologously co-expressed tagged KCNE2 and Testin proteins physically interact with one another, we do not yet know if Testin interacts with Kv1.5 itself, or whether its interaction with KCNE2 disrupts or alters physical interaction of Kv1.5 with KCNE2 (although co-expression of Testin reduces current density of Kv1.5-KCNE2, but not Kv1.5 channels, suggesting Testin retains some functional effects on the former). Therefore, we do not yet understand the mechanism by which Testin disrupts functional effects of KCNE2 on Kv1.5. Third, we do not know if Testin fails to alter effects of KCNE2 on KCNQ1 because the latter disrupts KCNE2-Testin interaction, or because Testin binds to a region of KCNE2 important for Kv1.5, but not KCNQ1, functional modulation. Fourth, we have not yet determined whether Testin binds to Kv1.5 but not KCNQ1, another possible mechanism to explain why Testin disrupts regulation by KCNE2 of Kv1.5 but not KCNQ1. Future biochemical and mutagenesis studies can be employed to address these mechanistic questions.
